# Safety and outcomes of thrombolytic therapy in patients with pulmonary embolism and thrombocytopenia: A systematic review

**DOI:** 10.5339/qmj.2022.33

**Published:** 2022-08-05

**Authors:** Fateen Ata, Wanis Hamad Ibrahim, Mohammad Nasser Affas, Haseeb Ahmad Khan, Hafiz Waqas Younas, Zakaria Maat, Sabah Elshayeb Ali Mohamed, Balqis Daoudi

**Affiliations:** ^1^Department of Internal Medicine, Hamad General Hospital, Hamad Medical Corporation, Doha, Qatar E-mail: docfateenata@gmail.com; E-mail: FAta@hamad.qa; ^2^Department of Pulmonology, Hamad General Hospital, Hamad Medical Corporation, Doha, Qatar; ^3^Department of Internal Medicine, Nishtar Hospital, Multan, Pakistan; ^4^Department of Emergency Medicine, Forth Valley Royal Hospital, Larbert, Scotland, UK; ^5^Clinical Pharmacy Department, Hamad Medical Corporation, Doha, Qatar

**Keywords:** Pulmonary embolism, PE, Thrombocytopenia, platelets, anticoagulation, thrombolysis

## Abstract

Thrombolysis is an established therapeutic modality for patients with high-risk (and some selected intermediate-risk) pulmonary embolism (PE) with hemodynamic instability. Physicians sometimes experience cases where both a high-risk PE and thrombocytopenia coexist. Although thrombocytopenia of <  100 × 10^3^/mm^3^ is considered a contraindication in patients with ischemic stroke, the safety and outcomes of thrombolysis in patients with acute PE and thrombocytopenia are unknown. This systemic review aimed to pool data on the safety and outcomes of thrombolysis use in patients with PE and platelet count less than 150 × 10^3^/mm^3^. Patients’ demographics, clinical characteristics, management, type of thrombolytic therapy, and outcomes were extracted and analyzed. Of 283 articles identified through the systematic search, 11 case reports fulfilled the inclusion criteria. The mean age of the patients was 52.27 years, and 54.5% were women. The median platelet level before thrombolysis was 65.50 × 10^3^/mm^3^. Before thrombolysis was initiated, the lowest and highest platelet levels were 29 × 10^3^/mm^3^ and 105 × 10^3^/mm^3^, respectively. Alteplase was used in 10 patients and urokinase in one patient. One patient who had a massive PE died of aspiration pneumonia. Interestingly, no thrombocytopenia-related complications were reported. This systematic review highlights the potential benefits and safety of thrombolysis in patients with acute PE in the context of thrombocytopenia. Nevertheless, data available in the literature concerning this topic are scarce and limited to case reports. More extensive studies on the use of thrombolysis in patients with PE and thrombocytopenia are desperately needed.

Systematic review registration: The protocol has been registered in the International Prospective Register of Systematic Reviews (PROSPERO): CRD42021286415.

## Introduction

Venous thromboembolism (VTE) remains a major contributor to worldwide mortality with a rising incidence. The current incidence of VTE is 1–2 cases/1000 persons per year in Europe and the USA.^1^ Pulmonary embolism (PE) is a severe emergency subset of VTE that warrants urgent diagnosis and management. The primary mechanism by which an acute PE can cause mortality lies in pulmonary vascular obstruction and constriction, which leads to right ventricular dysfunction. This may manifest as fatal arrhythmias and obstructive shock.^1^ Extensive research has been conducted on the management of acute PE. Established treatment modalities are anticoagulation (AC), thrombolysis, and surgical or percutaneous thrombectomy. Whether AC alone or AC with reperfusion therapy would suffice mainly depends on the risk stratification of the PE.^2-4^ Recent PE clinical guidelines stratify the risks and severity of acute PE based on its clinical presentation and hemodynamic effects. High-risk PE is defined by the presence of hemodynamic instability that includes one of the following clinical presentations: cardiac arrest, obstructive shock (systolic blood pressure < 90 mmHg despite an adequate filling status, in combination with end-organ hypoperfusion), or persistent hypotension (systolic blood pressure ≥ 40 mmHg for >15 min, not caused by new-onset arrhythmia, hypovolemia, or sepsis). Intermediate-risk PE is defined by the presence of right ventricular dysfunction (on echocardiography/radiologic imaging) and elevated cardiac biomarker levels in the circulation (particularly a positive cardiac troponin test). Low-risk PE is defined by the absence of high- or intermediate-risk features.^3^ Furthermore, the treatment modality depends on the expertise of the physician and treating center, patient presentation, and PE severity. Patients with hemodynamic instability secondary to PE usually require aggressive management with urgent thrombolysis with or without surgical management. A recent Cochrane review (which comprised randomized controlled trials comparing thrombolysis with AC versus AC alone or surgical removal) revealed that thrombolysis reduced the mortality in such patients compared with heparin alone (odds ratio (OR) 0.57, 95% confidence interval (CI) 0.37–0.87).^5^

Although still controversial, thrombolysis has shown mortality benefits in patients with intermediate-risk PE (without hypotension or shock but with right ventricular dysfunction).^6-8^ The primary concern when using thrombolytic therapy in intermediate- and high-risk PE is the risk of significant bleeding. Thrombolysis is increasingly being used because of its well-established efficacy in mortality reduction regarding intermediate–high to high-risk PE and easy availability of thrombolysis compared with other aggressive measures such as surgical removal or percutaneous thrombectomy. Methods used to perform thrombolysis in patients with PE include systemic thrombolysis, catheter-guided thrombolysis, and ultrasound-accelerated catheter-thrombolysis.^9,10^ Drugs approved for thrombolysis in patients with PE include streptokinase, urokinase, tenecteplase, reteplase, and alteplase.^10^ Among these, streptokinase and urokinase require loading doses, whereas others are given as boluses. Additionally, doses of alteplase lower than the recommended have been used successfully.^11^ Although thrombocytopenia of < 100 × 10^3^/mm^3^ is generally considered a contraindication in patients with ischemic stroke, some evidence suggests the safe use of thrombolysis with lower platelet levels in acute ischemic stroke.^12^ Furthermore, physicians sometimes experience cases where a high-risk PE and thrombocytopenia coexist, and surgical or catheter-induced thrombolysis is not feasible in their facility. To the best of our knowledge, the safety and outcomes of thrombolysis in the setting of acute PE and thrombocytopenia have not been addressed in a previous systematic review. In this review, we searched and pooled data from the available articles describing the use of thrombolysis in patients with PE who had thrombocytopenia, focusing on patients’ demographics, methods of thrombolysis used, complications, and outcomes.

## Materials And Methods

### Literature search

We searched systematically the databases of (from any date up to 20 October 2021) PubMed, Scopus, and Google Scholar for any English language articles pertinent to our research question using the following search terms:

“thrombocytopaenia”[All Fields] OR “thrombocytopenia”[MeSH Terms] OR “thrombocytopenia”[All Fields] OR “thrombocytopenias”[All Fields] OR (“low”[All Fields] AND (“blood platelets”[MeSH Terms] OR (“blood”[All Fields] AND “platelets”[All Fields]) OR “blood platelets”[All Fields] OR “platelet”[All Fields] OR “platelets”[All Fields] OR “platelet s”[All Fields] OR “plateletes”[All Fields])) AND Search: pulmonary embolism Sort by: Most Recent

“pulmonary embolism”[MeSH Terms] OR (“pulmonary”[All Fields] AND “embolism”[All Fields]) OR “pulmonary embolism”[All Fields] AND Search: ((((thrombolysis) OR (alteplase)) OR (reteplase)) OR (Tpa)) OR (rtpa) Sort by: Most Recent “thrombolysis”[All Fields] OR (“tissue plasminogen activator”[MeSH Terms] OR (“tissue”[All Fields] AND “plasminogen”[All Fields] AND “activator”[All Fields]) OR “tissue plasminogen activator”[All Fields] OR “alteplase”[All Fields]) OR (“reteplase”[Supplementary Concept] OR “reteplase”[All Fields]) OR “Tpa”[All Fields] OR “rtpa”[All Fields].

Our predefined PECO question was as follows:

### Population

Patients of any age with a radiologically confirmed diagnosis of PE and laboratory-confirmed thrombocytopenia (platelets <  150 × 10^3^/mm^3^) who underwent thrombolysis.

### Exposure

Thrombolysis

### Control

None

### Outcomes

Resolution, progression, persistence of PE, death, or complications of thrombolysis.

### Study selection and inclusion criteria

**Figure 1.**
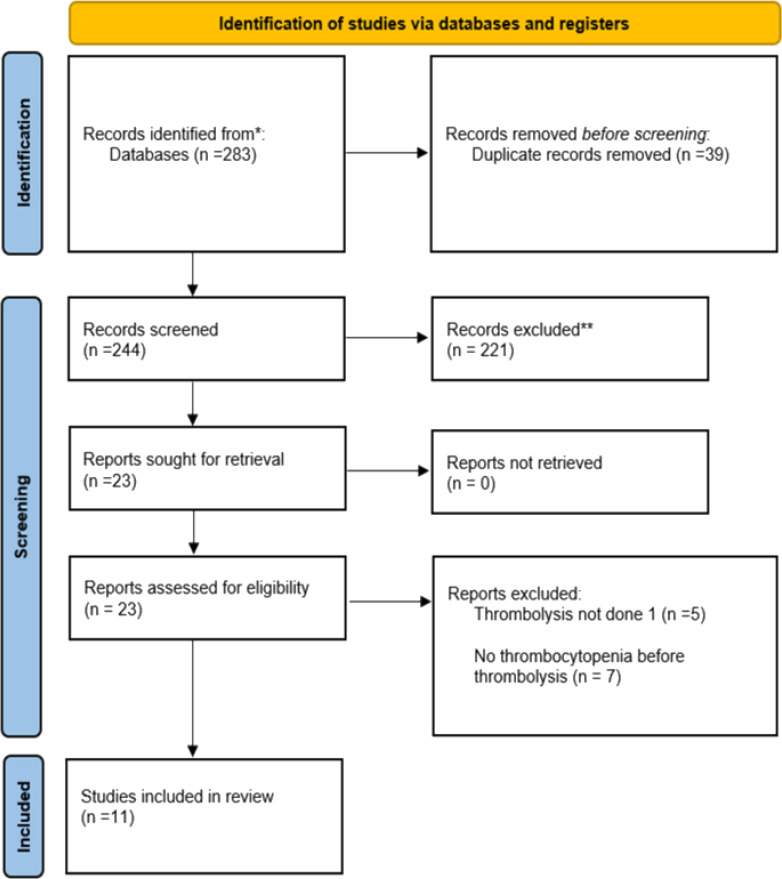
PRISMA flow diagram of identified, included, and excluded studies.

All article types, including case reports, case series, retrospective studies, prospective studies, and randomized studies addressing our research question, were considered eligible for inclusion. Endnote was used for the screening process of the articles that were found relevant to the topic. Two reviewers independently reviewed the extracted studies by title, abstract, and keywords. This was followed by a detailed full-length screening of the included studies. A third reviewer conducted an independent review of the disputed articles to reach an agreement. Figure 1 shows the PRISMA flow diagram that maps out the number of identified, included, and excluded records.

### Exclusion criteria

Articles published in languages other than English, articles on PE without radiologic confirmation, and articles on PE without laboratory confirmation of thrombocytopenia (platelets <  150 × 10^3^/mm^3^) were excluded.

### Bias assessment

Joanna Briggs Institute case report appraisal checklist for inclusion in systematic reviews was utilized to assess the quality of case reports and series.^13^

### Data collection and statistical analysis

Data on sociodemographic variables, clinical and radiological data, and provoking factors of PE were collected. We also recorded the values of platelets in these patients and thrombolysis outcomes. Data were captured and analyzed in Microsoft Excel 2020. Descriptive and summary statistics were used to describe the study cohort's sociodemographic parameters. Platelet levels were collected at three-time points: admission, lowest level during the hospital stay, and last platelet level before thrombolysis ensued. Continuous variables (means ±  standard deviation or median with interquartile range) and categorical variables (presented as numbers with percentages) were reported as appropriate.

## Results

Only 11 articles (all were case reports) that met the inclusion criteria of thrombolysis use in the context of PE and thrombocytopenia were found. One abstract that reported 17 patients with upper and lower limb deep vein thrombosis (DVT) and thrombocytopenia who underwent catheter-directed thrombolysis without thrombolysis-related complications was excluded because it did not include patients with PE and the required details about the cases.^14^

### Demographics and clinical features

Table 1 summarizes the patients’ demographics from the included articles (n = 11).^15-26^ The mean age at presentation was 52.27 ± 13.22 years, and 54.5% were women. The most commonly reported clinical findings were orthopnea (N 6, 54.5%) and lower limb pain (n = 3, 27.3%). Chest pain (n = 2, 18.2%) and hemodynamic instability with features of obstructive shock (n = 1, 9.1%) were less common. Only one patient with massive PE died from aspiration pneumonia. No thrombocytopenia-related mortality was reported.

### Epidemiology

A concomitant peripheral DVT was observed in 3 (27.3%) patients; one (9.1%) patient reported bilateral DVT, one (9.1%) distal, and one (9.1%) proximal. A history of previous DVT and PE was reported in 18.2% and 9.1% of the patients, respectively. The most common reported provoking factor for DVT/PE was malignancy (27.3%), followed by heparin-induced thrombocytopenia (HIT) (18.2%). Other less common provoking factors were also reported (Table 2).

### Comorbidities

Of all 11 cases, chronic lung disease and hypertension were reported in 2 (18.2%) and 1 (9.1%) cases, respectively. A previous history of PE was reported in only 1 (9.1%) patient. A history of cardiovascular disease (including coronary artery disease and heart failure) was found in 2 (18.2%) patients. A patent foramen ovale was found in 1 (9.1%) case, and atrial fibrillation was reported in 1 (9.1%) patient.

### Vital signs and laboratory data

Table 3 describes the vital signs and laboratory findings of the included patients. The D-dimer level was 6290 ng/mL in one patient. Five patients (45.5%) had platelet level between 100 and 149 × 10^3^/mm^3^ at presentation, four (36.4%) had levels between 50 and 99 × 10^3^/mm^3^, and two (18.2%) had levels between 20 and 49 × 10^3^/mm^3^ at presentation. A 56-year-old man who developed HIT had the lowest platelet level (before thrombolysis was initiated; 29 × 10^3^/mm^3^).^15^ A 13-day-old patient with bilateral PE and acute cor pulmonale had the highest platelet count just before thrombolysis (105 × 10^3^/mm^3^).^26^ Both patients completely recovered without thrombolysis-related complications.

### Diagnostics

Computed tomography pulmonary angiogram (CTPA) was the most commonly used diagnostic test (n = 10, 90.9%), whereas a ventilation–perfusion (V/Q) scan was performed in 1 (9.1%) case. About half of the patients (n = 6, 54.5%) had features of right heart strain on echocardiography. Electrocardiographic (ECG) findings were reported in 6 cases, with right ventricular strain in 4 (36.4%) cases. PE shown on CTPA were solitary clots in 4 (36.4%) cases, two clots in 3 (27.3%), and >3 clots in 3 (27.3%). They were segmental in 3 (27.3%) cases and subsegmental in 1 (9.1%). Four (36.4%) cases were reported as massive PE on CT scans.

### Management

All 11 patients received thrombolysis, whereas 10 (90.9) were reported to have also received AC. The most commonly used AC method was heparin initially, followed by warfarin in 2 (18.2%) patients or heparin only in 2 (18.2%). Other patients reported the use of argatroban (n = 1, 9.1%), bivalirudin, and fondaparinux (n = 1, 9.1%), initial heparin then switched to argatroban (n = 1, 9.1%), or phenprocoumon (n = 1, 9.1%) due to HIT. The reason for the initiation of thrombolysis despite thrombocytopenia was not reported in the majority of the cases. A clear reason was reported in only two cases. The first patient was a 65-year-old woman with metastatic breast cancer who had thrombocytopenia due to chemotherapy (60 × 10^3^/mm^3^). She was planned for catheter-guided or surgical embolectomy; however, due to the nonavailability of the former, thrombolysis was decided.^24^ The second patient was a 60-year-old man with pancreatic cancer. He developed thrombocytopenia due to HIT (77 × 10^3^/mm^3^). He was deemed unfit for surgery because of his advanced cancer stage; thus, thrombolysis was performed.^25^ Overall, the most common thrombolysis method was systemic (n = 7, 63.6%), whereas catheter-guided thrombolysis was used in 4 (36.4%) patients. The most common thrombolytic agents used were alteplase in 10 (90.9%) patients and urokinase in 1 (9.1%). One patient (9.1%) reported administration of thrombolysis as a bolus, whereas others reported infusion ranging from 2 hours (n = 2, 18.2%), 12 hours (n = 1, 9.1%), 24 hours (n = 3, 27.3%), or >48 hours (n = 2, 18.2%). None of the included articles reported platelet transfusion to optimize platelet count before thrombolysis.

### Outcome and Complications

Among the 11 included cases, 1 (9.1%) reported death as the final outcome. This patient had a massive PE and received alteplase. The cause of death was reported as aspiration pneumonia, which complicated his course of hospitalization. Nevertheless, the authors reported an initial improvement in the patient's condition after alteplase initiation.^25^ Four cases (36.4%) required intensive care unit admissions, and 3 (27.3%) reported endotracheal intubation. Clot resolution was documented in 5 (45.5%) patients. Interestingly, no thrombocytopenia-related complications were reported. Reported complications were early infection in 1 (9.1%) case and aspiration pneumonia in another (9.1%), which occurred late. Follow-up was conducted on 9 (81.8%) patients. The median hospital stay was 49 days, with a mean of 36.67 ± 25.81 days.

## Discussion

Although bleeding diathesis and active bleeding are absolute contraindications for thrombolysis, there is no agreement on the level of platelet count to consider an absolute contraindication.^3^ There are many situations in which thrombocytopenia and PE can coexist in clinical practice. Examples of such situations are patients with malignancies, thrombotic thrombocytopenic purpura (TTP), disseminated intravascular coagulation, autoimmune and connective tissue disorders. Cancer is a significant risk factor for PE and can cause thrombocytopenia because of the associated bone marrow suppression and adverse effects of chemotherapy.^27^ In the present review, 3 (27.2%) patients had malignancies.^17,24,25^ The first patient was a women with breast cancer.^24^ Her thrombocytopenia (60 × 10^3^/mm^3^) was attributed to chemotherapy. She received thrombolysis as PE treatment following deterioration of her condition. She received alteplase (100 mg over 2 hours) with half-dose heparin and recovered successfully without treatment complications. The second patient, a man with metastatic pancreatic cancer, developed HIT when he was diagnosed and managed for a PE. His platelet level dropped from 150 × 10^3^/mm^3^ to 77 × 10^3^/mm^3^. However, due to worsening PE, he was re-perfused via thrombolysis (was also considered unfit for surgery because of advanced malignancy). He received alteplase at 0.5 mg/h for 20 hours. Although the patient's symptoms improved and oxygen demands decreased post thrombolysis, he ultimately died of aspiration pneumonia on day 6.^25^ The third patient had gastric carcinoma complicated by PE and HIT (platelet level, 65 × 10^3^/mm^3^). He received 30 mg of alteplase as reperfusion therapy. The reason for thrombolysis, despite thrombocytopenia, was not mentioned in this case.^17^ Other than solid malignancies, patients with hematologic cancers are also at dual risk of thrombocytopenia (bone marrow involvement and cytotoxic effects of therapy) and thrombosis (due to cancer itself, endothelial damage secondary to chemotherapy, and asparaginase induced pro-thrombotic state).^28^ Very few studies have evaluated the effects of AC or thrombolysis in such patients. In one study, of 82 patients with malignancy and VTE who had anticoagulation, 31 (37.8%) developed bleeding. Nevertheless, significant bleeding was observed in 10.9%.^29^ Experts recommend full-dose AC in these patients when platelet levels are more than 50 × 10^3^/mm^3^ and reduced dose AC when less than 50 × 10^3^/mm^3^. AC is not an absolute contraindication in either case, and thrombocytopenia should be managed concomitantly if needed.^30^ Thrombocytopenia in the setting of an otherwise pro-thrombotic state may not be a significant bleeding diathesis, as it would be in the absence of a pro-thrombotic state such as malignancy. In the CLOT trial (676 patients with cancer and VTE who received either dalteparin or oral AC), 31 (4.5%) patients had a major bleeding event. Of these 31 patients, only two had thrombocytopenia.^31^

In the present review, we could also find situations (other than malignancy) in which patients had PE along with thrombocytopenia and an immediate thrombectomy was not feasible, or the patient was not suitable for surgical management. In these cases, authors used thrombolysis as a last resort in patients with hemodynamically deteriorating condition and massive PE. Zhu et al. described a patient with TTP who experienced an acute PE attack. The patient received thrombolysis during the resuscitation, as he suffered a cardiac arrest secondary to PE. The patient responded well to thrombolytic therapy with no further neurological complications.^23^ Pishgahi et al. reported a case of high-risk PE with contraindications to thrombolysis (thrombocytopenia and malignancy with brain metastasis). The patient could not undergo surgery (due to her deteriorating condition) and received thrombolysis instead, with complete recovery from PE and no bleeding complications.^24^ Valencia et al. discussed the case of a male infant who was referred for treatment of PE and right heart failure. His platelet count was 105 × 10^3^/mm^3^. The multidisciplinary team recommended a tPA for thrombolysis rather than a thrombectomy. The tPA therapy resulted in recovery without any bleeding complications.^26^ Wu et al. reported a case of systemic lupus erythematosus-linked protein-losing enteropathy that presented as PE with evidence of right ventricular failure and pulmonary hypertension. The thrombus was defragmented with a percutaneous catheter-based technique and lysed with alteplase. He was managed successfully and experienced no complications, despite a platelet count of 98 × 10^3^/mm^3^.^20^

These reports further emphasize that thrombolytic therapy can be life-saving and safe in patients with deteriorating condition, high-risk PE, and thrombocytopenia. The feasibility and wide availability of thrombolysis compared with surgical or percutaneous thrombectomy have also made it an attractive mode of therapy in patients with worsening PE and thrombocytopenia. Nevertheless, the need and necessity for thrombolysis amid contraindications requires an individualized case assessment with a benefit–risk consideration from the treating physician.

In the present review, some patients with HIT and PE underwent thrombolysis. HIT is a coagulation disorder that may complicate heparin therapy (more commonly with unfractionated heparin). The procoagulant state may occur before thrombocytopenia. Patients can develop serious thrombotic complications, especially in HIT type 2.^32^ Bhalla et al. and Schmidt et al. reported cases of patients who had undergone major surgery. They suffered from PE and had iatrogenic low platelet count with heparin therapy. In both cases, heparin administration was stopped, argatroban was started, and thrombolysis was used as rescue therapy to evade life-threatening hemodynamic instability. The follow-up revealed no further thrombosis or bleeding complications.^19,21^ Interestingly, the current coronavirus disease 2019 (COVID-19) pandemic appears to impose a considerable risk for thrombocytopenia and VTE (including PE). Recent evidence suggests that the severe acute respiratory syndrome coronavirus 2 interacts with different cellular molecules, including enzymes, endothelial cells, platelets, neutrophil extracellular traps, thrombin, and histones, resulting in endothelial damage, and formation of microthrombi.^33^ The increasing prevalence of HIT during the pandemic caused by increased heparin use as a VTE prophylaxis is also an issue.^32^ AC appears to be nonprotective in preventing VTE in these patients.^33^ Apart from COVID-19, all the mainstream mRNA vaccines against the virus (including mRNA-1273, BNT162b2, and AZD1222 COVID-19 vaccines) have been reported to cause thrombosis at one end and thrombocytopenia at the other (called vaccine-induced immune thrombotic thrombocytopenia).^34,35^ Given that the pandemic continues and mass vaccinations have been used, a considerable population is currently at risk of thrombosis and thrombocytopenia.^36^ Studying the safety of thrombolysis in such a population is interesting in formulating guidelines for this patient group.

The main strength of our review lies in its novelty and a detailed literature search (involving three databases that were searched systematically) to find cases in which thrombolysis was used in patients with PE who had thrombocytopenia. Our review has some limitations inherent for systematic reviews of rare conditions. First, all the included studies are case reports. Despite the extensive search, we could not find any higher level of evidence, resulting in a small sample size. However, we did not conduct any correlation statistics to maintain data accuracy. Second, publication bias is possible, as we could only add published cases. Third, we acknowledge that case reports based on adverse effects of treatment may focus only on significant side effects and ignore minor events. In view of this, the results of this review should be taken with caution until a higher level of evidence is generated.

## Conclusion

This systematic review highlights the potential benefits and safety of thrombolysis use in patients with acute PE in the context of thrombocytopenia, as both the initial treatment and last resort when other treatment options are not feasible. Nevertheless, data available in the literature concerning this topic are scarce and limited to case reports. More extensive studies on patients with PE and thrombocytopenia are urgently required to refine guidelines concerning thrombolysis as a preferred treatment or as a second-line option.

## Declarations

### Ethics approval and consent to participate

Private information from individuals will not be published. This systematic review does not involve endangering participant rights. Ethical approval is not required for this systematic review as only a secondary analysis of data already available in the electronic databases was conducted.

### Consent for publication

Not applicable

### Availability of data and materials

Data sharing not applicable.

### Competing interests

The authors declare that they have no competing interests.

### Funding

This study was not funded.

### Acknowledgments

None.

### Authors’ contributions

**FA:** Methodology, data collection, analysis and interpretation, literature review, manuscript preparation, critical review, and revisions in the manuscript.

**WI:** Conceptualization, supervision, methodology, critical review, and revisions in the manuscript.

**MA:** Literature review, data collection, data analysis, manuscript writing.

**HK:** Literature review, manuscript writing.

**HY, ZM, SM, BD:** Literature review, data collection.

All the authors reviewed and approved the final version of the manuscript.

## Figures and Tables

**Table 1:** Summary of the patients with thrombocytopenia and PE.

Characteristics	Results (n=11)

Mean age (years)	Mean: 52.27 ± 13.22

Sex	Males: 5 (45.5%)

	Females: 6 (54.5%)

Hospital stay (days)	Mean: 36.67 + 25.81

AC used	Heparin*: 5 (45.5%)

	Warfarin**: 2 (18.2%)

	Aragtroban: 2 (18.2%)

	Not mentioned: 2 (18.2%)

Thrombolytics used	tPA/rTPA/rPA: 10 (90.9%)

	UK: 1 (9.1%)

	Agent Not mentioned: 1 (9.1%)

Mortality outcomes	Recovered: 10 (90.9%)

	Died: 1 (9.1%)


*All types of heparin. **All types of warfarin. (tPA, tissue plasminogen activator; rTPA, recombinant tissue plasminogen activator; rPA, Reteplase; LMWH, low-molecular-weight heparin; UfH, unfractionated heparin; NOACS, novel oral anticoagulants; UK, urokinase)

**Table 2:** Summary of reported provoking factors for DVT and PE.

PE provoking factor	Frequency	Percent

**Malignancy**	3	27.3%

**HIT**	2	18.2%

**Surgery**	1	9.1%

**PICC line-induced RA thrombus**	1	9.1%

**Lupus-associated protein losing enteropathy**	1	9.1%

**May-Thurner syndrome**	1	9.1%

**Recent ICU admission**	1	9.1%

**TTP**	1	9.1%


(HIT, heparin-induced thrombocytopenia; PICC, peripherally inserted central catheter; TTP, thrombotic thrombocytopenic purpura)

**Table 3:** Vital signs and platelet levels.

Characteristics	Median	Mean	Std

**SBP at admission (mm hg)**	91	88.67	21.41

**HR at admission (bpm)**	125	133.67	18.53

**PLT at presentation (10^3^/mm^3^)**	98	145.09	139.09

**PLT before thrombolysis (10^3^/mm^3^)**	69	66.70	27.44

**Lowest PLT (10^3^/mm^3^)**	65.50	68.83	29.10


(SBP, systolic blood pressure; HR, heart rate; PLT, platelets)
